# Diverse Environmental Microbiota as a Tool to Augment Biodiversity in Urban Landscaping Materials

**DOI:** 10.3389/fmicb.2019.00536

**Published:** 2019-03-22

**Authors:** Nan Hui, Mira Grönroos, Marja I. Roslund, Anirudra Parajuli, Heli K. Vari, Laura Soininen, Olli H. Laitinen, Aki Sinkkonen, The ADELE Research Group

**Affiliations:** ^1^Nature-Based Solutions Research Group, Ecology and Environment Research Programme, University of Helsinki, Lahti, Finland; ^2^Faculty of Medicine and Health Technology, Tampere University, Tampere, Finland

**Keywords:** bacterial diversity, microbial inoculant, opportunistic pathogen, safety directive EN1177, safety sand, sand box sand, sieved sand, skin microbiota

## Abstract

Human activities typically lead to simplified urban diversity, which in turn reduces microbial exposure and increases the risk to urban dwellers from non-communicable diseases. To overcome this, we developed a microbial inoculant from forest and agricultural materials that resembles microbiota in organic soils. Three different sand materials (sieved, safety, and sandbox) commonly used in playgrounds and other public spaces were enriched with 5% of the inoculant. Skin microbiota on fingers (identified from bacterial 16S rDNA determined using Illumina MiSeq sequencing) was compared after touching non-enriched and microbial inoculant-enriched sands. Exposure to the non-enriched materials changed the skin bacterial community composition in distinct ways. When the inoculant was added to the materials, the overall shift in community composition was larger and the differences between different sand materials almost disappeared. Inoculant-enriched sand materials increased bacterial diversity and richness but did not affect evenness at the OTU level on skin. The Firmicutes/Bacteroidetes ratio was higher after touching inoculant-enriched compared to non-enriched sand materials. The relative abundance of opportunistic pathogens on skin was 40–50% before touching sand materials, but dropped to 14 and 4% after touching standard and inoculant-enriched sand materials, respectively. When individual genera were analyzed, *Pseudomonas* sp. and *Sphingomonas* sp. were more abundant after touching standard, non-enriched sand materials, while only the relative abundance of *Chryseobacterium* sp. increased after touching the inoculant-enriched materials. As *Chryseobacterium* is harmless for healthy persons, and as standard landscaping materials and normal skin contain genera that include severe pathogens, the inoculant-enriched materials can be considered safe. Microbial inoculants could be specifically created to increase the proportion of non-pathogenic bacterial taxa and minimize the transfer of pathogenic taxa. We recommend further study into the usability of inoculant-enriched materials and their effects on the bacterial community composition of human skin and on the immune response.

## Introduction

Disconnection of man from soil is a major problem in developed countries (Flandroy et al., 2018). Coupled with a significant increase in hygiene levels (UNICEF, 2015), it results in decreased numbers of diverse environmental microbiota in the everyday life of urban dwellers. Comparative studies made within developed countries (Stein et al., 2016; Lehtimäki et al., 2018) and between urbanized and rural populations (see Schnorr et al., 2014) indicate that exposure to diverse environmental microbiota in everyday life is key to reducing the risk for many non-communicable diseases (Hanski et al., 2012; Flandroy et al., 2018). As a result, the incidence of most immune-mediated diseases, such as Type 1 diabetes (Kondrashova et al., 2013), Crohn’s disease (Ng et al., 2013), multiple sclerosis (Browne et al., 2014), and IgE-mediated sensitization and atopy (Hanski et al., 2012), is several times higher in developed than developing countries (Kondrashova et al., 2013). According to the biodiversity hypothesis (Hanski et al., 2012), the main reason for this is that development of the human immune system needs exposure to diverse environmental microbiota to avoid disorientation toward recognizing endogenous human proteins (i.e., autoantigens) or harmless particles, such as pollen or food allergens (Haahtela et al., 2013). As both urbanization and hygiene levels are expected to increase globally in the foreseeable future, there is a good reason to assume that the high prevalence of immune-mediated diseases will persist in developed countries and become increasingly problematic in developing countries that have so far avoided this epidemic.

Skin bacterial diversity is lower among people who have an immune-mediated disease (Hanski et al., 2012; Flandroy et al., 2018). We recently showed that urbanization in a highly developed country is negatively associated with the microbial diversity of the dirt that adults carry indoors (Parajuli et al., 2018). We also found evidence that differences seen previously between urban dwellers and rural hunter-gatherers in intercontinental studies (e.g., Schnorr et al., 2014) are similar to those between healthy rural and urban Finns (Parajuli et al., 2018). Because of these findings, we tested how touching organic gardening materials affects the bacterial community of urban dwellers and we found that even brief contact with organic gardening materials increases and diversifies the skin bacterial community, and there is a large variation between materials (Grönroos et al., 2018). We subsequently developed a rich and diverse microbial inoculant of organic materials and exposed urban volunteers to this material three times a day for 2 weeks (Nurminen et al., 2018). The surprising result was that daily contacts with this microbial inoculant changed the stool bacterial community and influenced blood levels of transforming growth factor beta (TGF-β) (Nurminen et al., 2018). As TGF-β is an immunoregulatory cytokine, we hypothesize that adding the microbial inoculant to the everyday environment modifies the urban microbiome and potentially reduces the prevalence of severe immune-mediated diseases.

Along with beneficial effects, soil and dust exposure are known to cause diseases (Jeffery and van der Putten, 2011). In urban conditions, debris carried indoors contains higher relative abundance of certain bacterial genera known to contain pathogens than in rural conditions (Parajuli et al., 2018). In addition, non-communicable diseases may result from airborne inorganic and organic dust that drive inflammatory responses (Rylander, 1994; Schenker, 2010). Adverse effects of dust *per se* are typically related to occupational health, i.e., frequent long-term daily exposure to specific dust-bound particles or pathogens (Schenker, 2000). Adverse effects can be minimized using existing technologies and urban planning. Even though opportunistic pathogens are abundant in gardening and landscaping materials (Hui et al., 2017), they are not routinely screened. Utilization of diverse environmental microbiota in the prevention of immune-mediated diseases must be safe. Therefore, in the current study we screened for all known opportunistic and facultative bacterial pathogens on skin.

In parallel with studies on the urban microbiome, we have explored consumer preferences; it is useless to develop solutions not accepted by consumers. We observed that radical innovations like touching a soil-like material are not preferred by consumers (Puhakka et al., 2018b), but instead, they are more willing to accept familiar products with an immunomodulatory twist (Puhakka et al., 2018a). Therefore, the logical step is to perform a study in which urban dwellers are exposed to a microbiologically diverse inoculant that is scalable for manufacturing techniques. As our studies revealed very low bacterial abundances in landscaping materials made of mineral soil particles, such as sand and gravel products (Supplementary Table S1), these materials were selected to be modified by combining with the microbiologically diverse inoculant developed in our laboratory. The purpose was to test how the skin microbiome of urban dwellers is altered when exposed to modified versus traditional landscaping materials. We hypothesized that (1) skin microbiota on fingers differs among participants, (2) each landscaping material changes the microbiota in a unique way, (3) touching modified landscaping materials changes the microbiota in a unique way that hides differences between different landscaping materials, and (4) touching modified landscaping materials decreases the abundance of potential pathogens on skin.

## Materials and Methods

### Experimental Design

Three different mineral soil materials were received from Rudus Oy (Renkomäen kiviainesmyynti, Lahti, Finland): sieved sand (<8 mm), safety sand used in children’s playgrounds (1–8 mm) and sandbox sand (<4 mm), hereafter, jointly referred to as sands. These sands are commonly used as surface soil materials in public parks, schools and kindergartens. Each material was divided in two parts. The first part was used unaltered, and the second part was mixed with freeze-dried organic material (ratio 1:20). This organic material is the microbiologically rich and diverse mixture of plant and soil-based materials (hereafter referred to as the microbial inoculant or the inoculant) previously described and tested by Nurminen et al. (2018). In short, the microbial inoculant contained sieved composted materials comprising crushed tree bark and mulch, dung, deciduous leaf litter, peat and agricultural sludge, and dried, crushed and sieved *Sphagnum* moss. The inoculant was saturated with ultra-pure mQ water and kept in the laminar for 4 h, covered with a lid but with holes in the side walls of the container to allow adequate air circulation. The soil–water mixture was then hand-squeezed using sterilized laboratory gloves over an ethanol-cleaned 250 μm sieve placed above another sterile plastic container. The extract was collected in separate 50 ml Falcon tubes, frozen at -20°C overnight and freeze-dried for 48 h (Christ Alpha 1–4, B.Braun Biotech International).

Five urban volunteers (healthy urban office workers, age 30–50, three females, two males) tested the three sand materials with and without the microbial inoculant in July 2017. First, volunteers washed their hands with soap in tap water for 20 s and patted their hands dry with a towel. Next, an assistant took skin swab samples from two fingers, identically from both hands, to collect the before samples. Each finger was stroked five times from the tip to the lowest joint with a sterile nylon swab (Copan Diagnostics, Murrieta, CA, United States) dipped in sterile 0.15M NaCl+ 0.1% Tween 20 solution. Volunteers then rubbed one of the fingers in sand for 20 s and the other finger in the same material mixed with the microbial inoculant (5% inoculant, 95% sand). The assistant took the second round of skin swab samples, the after samples, 5 min after participants washed their hands. The procedure was repeated for all test materials separately and materials were tested in a random order. No more than one material was tested on any day and there was at least a 1-day interval between the tests. Four persons touched each mineral soil material. Fingers and persons were randomized. Skin swab samples were stored in a sterile polyethene tube in a deep freezer (<-70°C) until the DNA extraction.

DNA was extracted from the samples using the Fast DNA spin kit for soil (MP biomedicals, Santa Ana, CA, United States) following manufacturer’s instructions. DNA samples were prepared for sequencing as in Grönroos et al. (2018). In brief, highly variable regions (V3 and V4) of the bacterial 16S rRNA gene were amplified using the primers 515F 5′- GTGCCAGCMGCCGCGGTAA-3′ and 806R 5′- GGACTACHVGGGTWTCTAAT-3′ with truncated Illumina overhangs. In the secondary PCR, the full-length P5 adapter and Indexed P7 adapters were used. The PCR was performed as described by Parajuli et al. (2018). The obtained PCR products were cleaned using AMPure XP (Agencourt, Beckman-Coulter, United States) and quantitated using Qubit (Invitrogen, United States). The DNA samples were analyzed using the Fragment Analyzer (Advanced Analytical, United States) and the amplicons were sequenced as paired-end (300 bp + 300 bp) in FIMM Seq Lab (Helsinki, Finland) with Illumina MiSeq and v3 reagent kit. The paired fastq files are available in the Sequence Read Archive at NCBI ^1^ under accession number SAMN10371562 – SAMN10371609.

Ethical approval was received from the ethics committee of Tampere University Hospital (case number: ETL R15081). The study was carried out in accordance with the relevant guidelines and regulations in Finland and the volunteers signed informed consents.

### Bioinformatics

Paired-end sequence data (.fastq) were processed using mothur version 1.35.1 (Schloss et al., 2009). Bacterial.fastq files were contiged and sequences were removed that had any ambiguous bases, more than one mismatch in the primers, homopolymers longer than 8 bp, or an overlap shorter than 50 bp.

Bacterial sequences were aligned against a SILVA reference database, preclustered to remove erroneous reads (Huse et al., 2010), and screened for chimeras with the Usearch algorithm (Edgar et al., 2011). Non-chimeric sequences were assigned to taxa using the Naïve Bayesian Classifier (Wang et al., 2007) against the RDP training set (version 10). Non-target sequences (mitochondria, chloroplast, Archaea) were removed. Sequences were clustered to OTUs at 97% similarity using nearest neighbor joining (single linkage) that conservatively assigns sequences to OTUs.

If the abundance of an OTU was ≤10 sequences across all experimental units, it was excluded from statistical analysis as most of the low-abundance OTUs are PCR or sequencing artifacts (Tedersoo et al., 2010; Brown et al., 2015; Oliver et al., 2015). We estimated richness and diversity metrics for bacterial communities in mothur. Observed OTU richness (Sobs), the complement of Simpson’s diversity (1/D: 1/Σpi2), and Simpson’s evenness (ED: 1/Σpi2/S), with pi representing frequency of each OTU within a sample, were iteratively calculated and subsampled at 2558 sequences per sample.

### Identification of Potential Human Pathogens

To identify potential human pathogens in our dataset, we used a list of bacterial genera that include potentially pathogenic species (opportunistic + facultative) published in Appendix A by Taylor et al. (2001). For each of our samples, we counted the number of genera listed in Taylor et al. (2001). We also counted the collective relative abundance of sequences belonging to these genera and identified potentially pathogenic genera with relative abundance >1%. The threshold was selected because the number of OTUs per sample ranged from 266 to 3766 in the after samples, and the purpose was to compare genera statistically. Finally, we compared before samples with after samples, and samples taken after touching sand materials mixed with the microbial inoculant to those taken after touching non-enriched sand materials.

OTU data was inserted to KEGG: Kyoto Encyclopedia of Genes and Genomes^2^ to analyze pathways associated with OTUs on skin (Kanehisa and Goto, 2000) following standard protocol, with a particular emphasis on searching for pathogen associations. The functionality of bacterial communities was predicted using the PICRUSt software (Langille et al., 2013), which predicts functional pathways from the 16S rRNA reads. First, an OTU table in biom format was obtained from the filtered reads by using the mothur program and by querying the data against a reference collection (GreenGenes database, May 2013 version^3^). The resulting OTU biom table was then used for microbial community metagenome prediction with PICRUSt on the online Galaxy interface^4^. PICRUSt was then used to derive relative Kyoto Encyclopedia of Genes and Genomes (KEGG) Pathway benchmarks.

### Statistical Analyses

All statistical analyses were performed in R (version 3.2.1, R Development Core Team, 2018). To analyze differences in the after samples between the microbial inoculant treatment and the mineral soil treatment, we used generalized linear mixed models (GLMM), with the lmer function in the lme4 package, to compare diversity indices, relative abundance of major phyla, four classes within Proteobacteria, and the nine most abundant genera. The relative abundance and number of genera containing human pathogens were compared using one-way ANOVA. The response variables were Ln-transformed to approximate normality. Since skin swab samples were taken from five study persons, study person was added as a random factor in the models. Non-metric multidimensional scaling (NMDS) analyses were performed using the vegan package to visualize the bacterial communities. Permutation tests were applied on the community structures by fitting procedure (the *envfit* function in the vegan package) and the Bray-Curtis coefficient was used as the dissimilarity measure.

## Results

### Microbial Inoculant Exposure Increases Skin Bacterial Richness and Diversity

**FIGURE 1:**
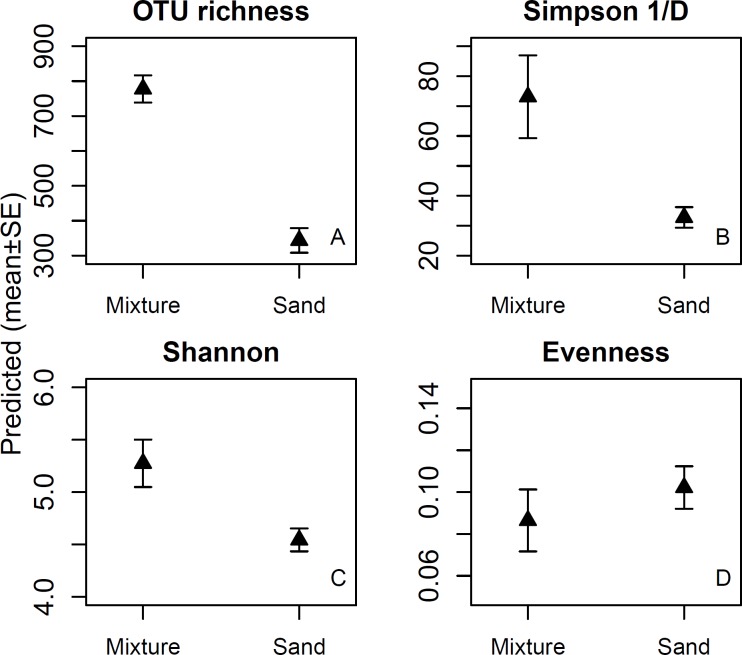
OTU richness (1A), Simpson 1/D (1B) and Shannon indices (1C), and evenness of skin bacterial communities (1D) after touching sand materials (sand) or the same sand materials containing 5% microbial inoculant (mixture). Details in Experimental design.

In the before samples, bacterial OTU richness ranged between 57 and 130, Simpson 1/D between 2.42 and 3.730, Shannon index between 1.99 and 7.62, and evenness between 0.07 and 0.11. Exposure to the microbial inoculant significantly increased the diversity indices, but not evenness (Table 1). After the exposure, bacterial OTU richness, Simpson 1/D and Shannon indices were higher for the inoculant-enriched sand treatments than for the non-enriched mineral sand treatments, but evenness remained unchanged (Figure 1 and Table 1).

**Table 1:** GLMM results of the bacterial diversity and relative abundance of taxa.

		Intercept	Mixture	SE	Prob > | t|
**Before vs. after (all samples included)**					
Sobs		332.083	228.542	26.220	0.000
Shannon		4.090	0.819	0.082	0.000
Simpson		33.621	19.316	4.103	0.000
Evenness		0.117	-0.012	0.006	0.055
**Mixture vs. control (after samples only)**					
Sobs		560.625	216.958	26.333	0.000
Shannon		4.909	0.366	0.126	0.008
Simpson		52.937	20.158	7.117	0.010
Evenness		0.094	-0.008	0.009	0.390
*Planomicrobium*		0.044	0.043	0.013	0.004
*Nocardioides*		0.019	-0.005	0.004	0.155
*Caldalkalibacillus*		0.019	-0.019	0.008	0.028
*Pseudomonas*		0.017	-0.014	0.007	0.051
*Marmoricola*		0.017	-0.016	0.004	0.000
*Luteimonas*		0.012	0.011	0.002	0.000
*Conexibacter*		0.010	-0.009	0.002	0.000
*Ralstonia*		0.010	-0.009	0.002	0.000
*Staphylococcus*		0.009	-0.008	0.002	0.002
*Polaromonas*		0.009	-0.009	0.004	0.033
Alphaproteobacteria		0.075	-0.013	0.005	0.011
Betaproteobacteria		0.091	-0.070	0.014	0.000
Deltaproteobacteria		0.010	0.003	0.001	0.007
Gammaproteobacteria		0.071	-0.001	0.011	0.897
Acidobacteria		0.034	-0.016	0.005	0.005
Actinobacteria		0.298	-0.104	0.022	0.000
Bacteroidetes		0.048	0.010	0.006	0.108
Firmicutes		0.212	0.131	0.030	0.000
Proteobacteria		0.252	-0.082	0.025	0.003


**FIGURE 2:**
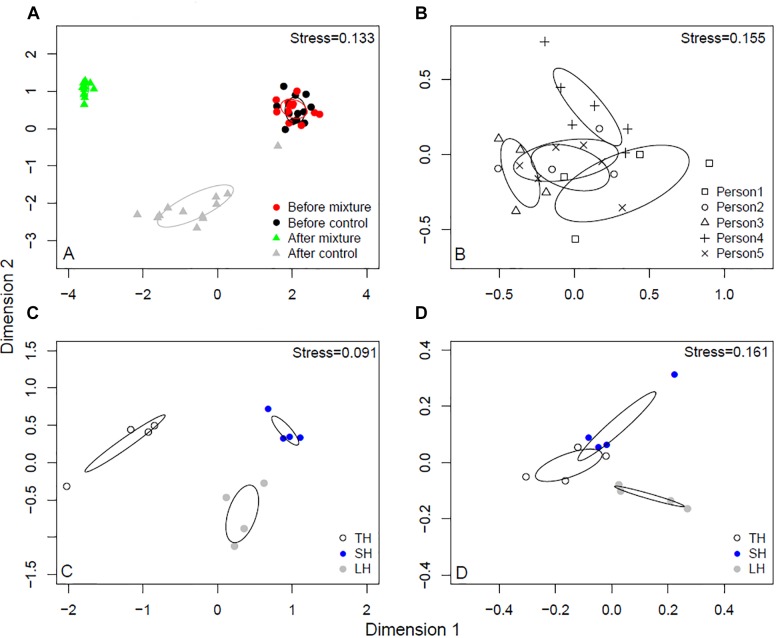
NMDS analyses of skin bacterial communities. **(A)** Before and after touching sand materials and sand materials containing 5% microbial inoculant (details in Experimental design). **(B)** The original skin bacterial communities of different study persons. **(C)** Skin bacterial communities after touching three different sands. **(D)** Skin bacterial communities after touching three different sands containing 5% microbial inoculant. TH, safety sand; SH, sieved sand; LH, sand box sand.

The before samples, after samples treated with non-enriched sands, and after samples with microbial inoculant-enriched sands, had distinct skin microbial communities (*r*^2^ = 0.722, *p* < 0.001; Figure 2A). The biggest shift occurred after touching sands enriched with the microbial inoculant (see *x*-axis in Figure 2A). The study participants originally had different skin bacterial communities (*r*^2^ = 0.68, *p* < 0.001; Figure 2B). In the after samples, touching sands enriched with the microbial inoculant changed the skin bacterial community in a unique way compared to the effect of touching non-enriched sands (*r*^2^ = 0.536, *p* = 0.002; Figure 2). Each type of sand led to unique changes in skin bacterial communities (*r*^2^ = 0.61, *p* = 0.006, Figure 2C). Some of these differences between sands still existed after using the microbial inoculant-enriched sands (*r*^2^ = 0.44, *p* = 0.011; Figure 2D).

### Taxonomic Shifts in Skin Bacteria After Exposure

**FIGURE 3:**
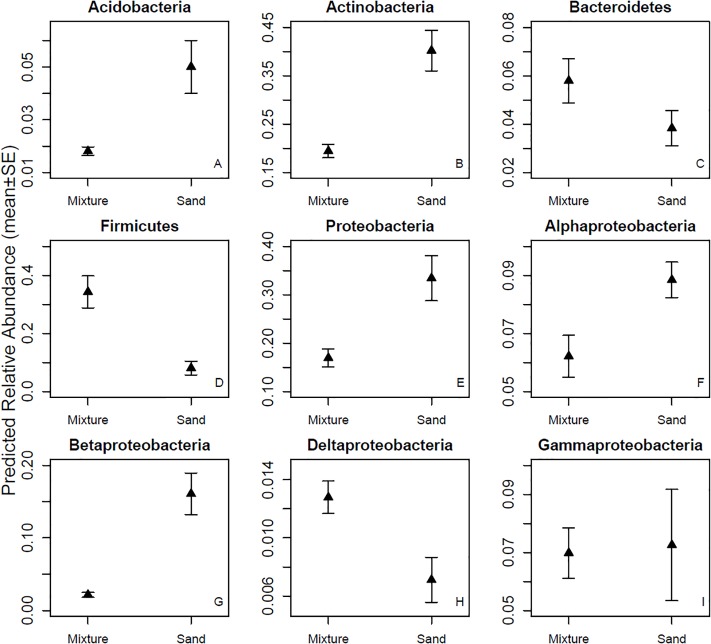
Relative abundances of major phyla and four classes. Skin bacterial communities were sampled after touching sand materials containing 5% microbial inoculant (mixture) or pure sand materials (sand). Details in Experimental design.

The bacterial OTUs in the skin swab samples were classified into 26 phyla. Firmicutes was the dominant (34.3%) phylum after touching the microbial inoculant-enriched sand materials, while Actinobacteria (41.7%) was the most abundant bacterial phylum after touching non-enriched sand materials. Among the five most common bacterial phyla, the relative abundance of Firmicutes was higher and the relative abundances of Acidobacteria, Actinobacteria, and Proteobacteria were lower in the inoculant-enriched samples compared to the non-enriched sand samples (Figure 3 and Table 1). Within Proteobacteria, touching microbial inoculant-enriched sand materials increased the relative abundance of Deltaproteobacteria, had no effect on the relative abundance of Gammaproteobacteria but decreased the relative abundance of Alfa- and Betaproteobacteria, as compared to skin bacterial communities after touching non-enriched sand materials (Figure 3 and Table 1).

**FIGURE 4:**
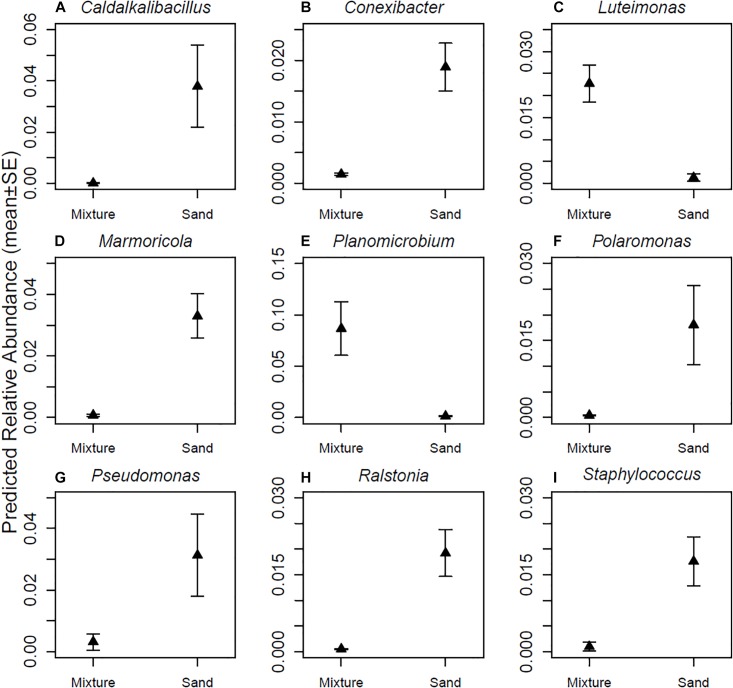
Relative abundances of major genera. **(A)** *Caldalkalibacillus* sp., **(B)** *Conexibacter* sp., **(C)** *Luteimonas* sp., **(D)** *Marmoricola* sp., **(E)** *Planomicrobium* sp., **(F)** *Polarimonas* sp., **(G)** *Pseudomonas* sp., **(H)** *Ralstonia* sp., and **(I)** *Staphylococcus* sp. Skin bacterial communities were sampled after touching sand materials containing 5% microbial inoculant (mixture) or pure sand materials (sand). Details in Experimental design.

The relative abundances of seven (*Caldalkalibacillus, Conexibacter, Marmoricola, Plaromonas, Pseudomonas, Ralstonia*, and *Staphylococcus*) out of the nine most abundant bacterial genera were higher in skin samples taken after touching non-modified sand materials as compared to samples taken after touching inoculant-enriched sand materials (Figure 4 and Table 1). The relative abundances of *Luteimonas* and *Planomicrobium* – the remaining two from the nine most abundant genera – were higher in samples taken after touching the inoculant-enriched sand materials (Figure 4 and Table 1).

### Genera Containing Potentially Pathogenic Species

The relative abundance of bacterial genera containing either opportunistic or facultative pathogens was 40–50% in hands washed with soap and water, i.e., in the before samples (Table 2). The relative abundance of the genera dropped to 14% after touching sand materials, and to 4% after touching sand materials mixed with the microbial inoculant (Table 2). At the same time, the number of genera containing potentially pathogenic species was approximately 5 percentage units higher in samples taken after touching microbial inoculant-enriched sands compared to other groups (Table 2). *Acinetobacter, Burkholderia, Corynebacterium, Ralstonia, Rothia*, and *Staphylococcus* had higher relative abundances in the before samples than in the after samples, and the relative abundances were typically lowest after touching microbial inoculant-enriched sand materials (Table 2). Relative abundances of *Pseudomonas* and *Sphingomonas* were high on skin only after study subjects touched non-enriched sand materials (Table 2). The relative abundance of only one genus containing potentially pathogenic species, *Chryseobacterium*, peaked after touching inoculant-enriched sand materials.

**Table 2:** Relative abundances of bacterial genera containing facultative or opportunistic human pathogens from skin before and after contact with microbial inoculant-enriched and non-enriched sand.

	Before sand + microbial inoculant	Before sand	After sand + microbial inoculant	After sand
Relative abundance of sequences (%)	45.6 ± 2.7^a^	43.3 ± 2.6^a^	4.2 ± 0.7^c^	13.6 ± 2.0^b^
Number of genera	17.1 ± 1.7^b^	19.4 ± 1.4^b^	23.8 ± 0.9^a^	16.9 ± 1.0^b^
Genera relative abundance > 1%				
*Acinetobacter*	2.7 ± 0.48	3.47 ± 0.44		
*Burkholderia*	13.24 ± 1.60	9.53 ± 1.03		1.41 ± 0.33
*Chryseobacterium*			1.43 ± 0.21	
*Corynebacterium*	3.98 ± 1.20	6.65 ± 1.73		
*Pseudomonas*				3.13 ± 1.34
*Ralstonia*	7.94 ± 1.38	5.27 ± 0.57		1.92 ± 0.46
*Rothia*	2.24 ± 1.92	1.03 ± 0.34		
*Sphingomonas*				1.57 ± 0.31
*Staphylococcus*	9.80 ± 2.23	10.83 ± 1.91		1.76 ± 0.48


*Mean ± SE. Superscript letters indicate significant differences in Tukey’s HSD test (*p* < 0.05). Empty cells represent relative abundance < 1%.*

### KEGG Analysis

Genes associated with *Staphylococcus* were associated with samples taken after touching microbial inoculant-enriched sand materials (Supplementary Table S2). No other associations with potential bacterial soil pathogens were found (Supplementary Tables S1, S2). Instead, samples taken after touching microbial inoculant-enriched sand materials had higher frequency of bacteria associated with several non-communicable diseases, viral and Eukaryotic infections and facultative pathogens that do not exist in soil (Supplementary Table S2).

## Discussion

The current study had four hypotheses in the context of modifying the microbiome of urban dwellers. The results confirmed the hypotheses that individual differences exist in skin microbial communities in hands, and that exposure-induced changes in skin microbial community composition depend on mineral soil type. The third hypothesis, on the contrary, was not supported as skin microbiota differences were small but still existed after mixing the sand materials with the microbial inoculant. The fourth hypothesis, proposing that the abundance of pathogens decreases after touching landscaping materials enriched with the microbial inoculant, was supported, and is discussed in detail hereafter. While individual differences of host-microbiome compositions affected the outcome of inoculant-enriched materials, the studied materials altered skin microbiome composition of all volunteers (see e.g., the right-left transition in Figure 2A). Even though many factors can be hypothesized to affect the ability of a host’s microbiome to resist or permit invasion and recolonization by bacteria in inoculant-enriched or non-enriched sands, the importance of factors like host health and personal hygiene had a minor role under the standardized conditions of the current study.

The highest relative abundances of individual genera were found after touching pure, non-enriched sand materials. The plausible reason is that the greater bacterial diversity and similar evenness reduced the relative abundances of major genera in the inoculant-enriched sand materials (see Figure 1). This indicates that numerous relatively rare OTUs from the inoculant were transferred to skin. This in turn indicates that most consequences attributable to single bacterial genus or species are likely to be more pronounced after touching pure non-enriched sand, as compared to touching microbial inoculant-enriched sand. As the consequence is often an infection related to an illness, the high diversity and richness in inoculant-enriched sand materials may help to minimize negative outcomes.

Our study demonstrates that adding nature-derived microbiota to sand landscaping materials enriches and increases uniformity in the skin microbial community composition after direct contact. Although we did not measure long-term effects of the short-term exposure, our previous study showed that daily indoor exposure to a biodiverse microbial inoculant can change the stool bacterial community in 2-weeks, probably caused by changes in the skin microbiota (Nurminen et al., 2018). As the tested materials are meant for everyday use, it is important to test whether microbiome modulation post-inoculated material contact and post-handwashing is strong enough to cause long-lasting major compositional skin microbiome changes. If proven to last for long periods, the microbial inoculant tested in this paper may affect immune response and reduce the risk of immune-mediated diseases. In this study, abundance shifts at the phylum level were complex: after touching pure non-enriched sand materials, the relative abundance of Actinobacteria, Acidobacteria and Proteobacteria was higher, whereas the relative abundance of Firmicutes and Bacteroidetes was lower compared to samples taken after touching inoculant-enriched materials (Figure 3). Interestingly, the Firmicutes/Bacteroidetes ratio was two-fold higher after touching the enriched sand materials compare to after touching non-enriched sand materials (see Figure 3). As a high Firmicutes/Bacteroidetes ratio is linked to a reduced obesity risk, the simple method represented in this study may have applicable value in combatting immune-mediated diseases.

The results indicate that transient skin bacterial communities on clean hands change drastically when a person is exposed to sand materials. Importantly, the frequency of genera containing opportunistic pathogens dropped to a fraction from the original state after touching microbial inoculant-enriched materials. In contrast, the relative abundance of *Sphingomonas*, a genus containing members that cause severe but not lethal infections in hospitals (Ryan and Adley, 2010), was higher after touching pure sand materials. Furthermore, *Pseudomonas* sp., which contains *P. aeruginosa* and other severe pathogens, increased after touching the pure, non-enriched sand materials. These materials are commonly used in kindergartens and playgrounds. One of the materials, safety sand, is one of the few products that fulfills safety directive EN1177 of the European Union. Our finding thus opens an alternative view to safety: should the safety directive be redirected to consider infectious diseases in parallel with safety issues related to consequences of physical injury.

*Luteimonas* and *Planomicrobium* were the only major genera that became more abundant on skin after touching microbial inoculant-enriched sand materials compared with touching pure, non-enriched sand materials. These two genera typically live in organic soil. This result indicates that sand materials enriched with the microbial inoculant transfer bacteria onto human skin. Numerous widespread soil bacteria belong to the same genera as human pathogens. Some human or animal pathogens found among soil and water-borne bacteria may be harmful to only one or a very narrow group of hosts, whereas their variants or ancestors may be totally non-harmful for the same hosts (Aujoulat et al., 2012; Taylor-Mulneix et al., 2017). Instead of specific recognition of each bacterial species, the human immune system’s pattern recognition receptors recognize pathogen-associated molecular patterns. These patterns are found both in pathogens and other, non-pathogenic, bacteria (Fawkner-Corbett et al., 2017). Therefore, taxonomically related non-pathogenic bacteria can activate an immune response without causing any disease. KEGG results support this kind of reasoning in the context of the current study, as the bacterial genes associated with *Staphylococcus* were abundant after touching inoculant-enriched sand materials even though *Staphylococcus* sp. itself was very rare in the samples (see Table 2). If the inoculant exposes humans to the *Staphylococcus* genus in general, the genus should be widespread after touching imoculant-enriched sand materials, enabling partial protection against *S. aureus* infection.

Since natural soils typically contain pathogens (Nieder et al., 2018), there is a risk that they will be introduced to humans via exposure to nature-derived microbiota. We used two separate methods to estimate infection risks in this study. Both methods, the one developed by us and the KEGG method commonly in use in medical studies (Langille et al., 2013), indicated that such risk seems to be only minor. Despite this, neither of the methods is perfect. Our method, that screens genera containing potentially pathogenic species, could instead be performed at a species level or across all taxa, instead of utilizing it only at the genus level as we did. Such improvement could aid in avoiding some false alarms. However, our method revealed that the only potentially pathogenic genus with >1% abundance in the microbial inoculant-enriched material was *Chryseobacterium* sp. This comprises two known opportunistic pathogens, *C. indologenes* and *C. hominis*, which have not been found to infect healthy people. *C. indologenes* rarely infects patients that use a ventilator system and sometimes infects severely immunocompromised and hospitalized infants (Olbrich et al., 2014). *C. hominis*, an even rarer pathogen, has only been found sporadically in clinical samples, such as in aortic valve and dialysis fluid (Vaneechoutte et al., 2007). As standard sand materials and even normal skin microflora may cause a threat to these patients, e.g., due to the high relative abundance of *Pseudomonas*, and as KEGG did not detect *Chryseobacterium*-related genes, the use of the immunomodulatory inoculant in landscaping materials has turned out to be a sophisticated strategy to increase richness and diversity on skin. Particularly, if the results of the current study are combined with those in our previous studies (Sinkkonen et al., 2013a,b; Parajuli et al., 2017; Mikkonen et al., 2018; Sinkkonen et al., 2018; Roslund et al., 2018), biodiverse surface soil materials may be suitable to balance human-induced disturbances in urban environments. The suitability of sand materials enriched with the immunomodulatory inoculant should be studied at a larger scale and over a longer time, with comparisons of different recipes of microbial inoculants. If the results of such a study are encouraging, the effects of the inoculant-enriched materials on skin and stool bacterial community composition, and ultimately immune response, should be investigated further. The goal of these potential upcoming studies will be to develop urban landscaping materials, the exposure to which would strengthen immunoregulatory pathways and reduce reactivity toward autoantigens and harmless immunogens such as pollen, food and animal scurf, but still permit the immune system’s reactions toward pathogens.

## Members of the Adele Research Group

Mira Grönroos, Nan Hui, Olli H. Laitinen, Raul Kalvo, Noora Nurminen, Sami Oikarinen, Anirudra Parajuli, Riikka Puhakka, Marja I. Roslund, Laura Soininen, Heli K. Vari, Guoyong Yan, Juho Rajaniemi, Heikki Hyöty, and Aki Sinkkonen.

## Data Availability

The datasets generated for this study can be found in Sequence Read Archive, SRP115276.

## Author Contributions

AS designed the study jointly with MG and MR. MG, OL, and AS developed the pathogen analysis method. NH analyzed the data statistically and wrote the first draft of Results, Bioinformatics, and Statistical Analysis. AS wrote Introduction and Discussion and the terminology in Results. MR wrote the Experimental design. MG wrote the pathogen and qPCR methods. AP and HV manufactured the microbial inoculant. MG, OL, MR, AS, and LS finalized the manuscript.

## Conflict of Interest Statement

MG, AP, MR, AS, and HV are the inventors in a patent application submitted by University of Helsinki (Patent application number 175196 at Finnish Patent and Registration Office). The remaining authors declare that the research was conducted in the absence of any commercial or financial relationships that could be construed as a potential conflict of interest.

## Footnotes

1 www.ncbi.nlm.nih.gov

2 https://www.genome.jp/kegg/

3 http://greengenes.lbl.gov

4 http://huttenhower.sph.harvard.edu/galaxy/
