# Supplementary material for: Diverse Environmental Microbiota as a Tool to Augment Biodiversity in Urban Landscaping Materials
Source: Front Microbiol. 2019 Mar 22;10:536. doi: 10.3389/fmicb.2019.00536 (PMC6438878; doi:10.3389/fmicb.2019.00536)
Supplement: Supplementary file 1 [file Table_1.DOCX]

Supplementary Material

Diverse environmental microbiota as a tool to augment biodiversity in urban landscaping materials

Hui Nan, Mira Grönroos, Marja I. Roslund, Anirudra Parajuli, Heli K. Vari, Laura Soininen, Olli Laitinen, Aki Sinkkonen* and the ADELE research group (Heikki Hyöty, Raul Kalvo, Noora Nurminen, Sami Oikarinen, Riikka Puhakka, Juho Rajaniemi)

*** Correspondence:** Corresponding Author: aki.sinkkonen@helsinki.fi

# Preliminary survey of bacterial abundance

## Methods

# We conducted a preliminary survey to compare bacterial abundance in different kind of soil materials. We tested four types of soil materials: sand/gravel from aggregate producer, organic soil, natural forest soil and freeze-dried extract. For sand/gravel samples, five different materials especially used in planning of urban spaces e.g. outdoor areas of daycare centers were received from an aggregate producer (Rudus Oy, Finland). For organic soils, fourteen organic soil materials, including commercially available soil products and semi-finished products used for manufacturing such soil products (e.g. leaf compost) were sampled. For natural forest soils, wet and moist habitat types were sampled. Freeze-dried extract was produced similarly as the diversity blend that was used in the experiment described in the main paper. Sampling was done with a sterile polyethene spoon and the sample was frozen in a clean polyethene freezer bag. For all samples, 2 g of soil was collected and the samples were stored in deep freezer (<-70°C) before DNA extraction. Approximately 0.25 g soil was used for DNA extraction. Total DNA was extracted from samples using PowerSoil® DNA Isolation Kit (MoBio Laboratories, Inc., Carlsbad, CA, USA) according to the manufacturer’s standard protocol. Two organic soil samples were extracted using PowerMax® DNA Isolation Kit (one sample from each of organic soils and one sample of natural forest soils).

# The quantitative PCRs of bacterial 16S rDNA were based on SYBR green detection. PCRs were carried out with the Light Cycler 96 Quantitative real-time PCR machine (MJ Research, MA, USA) with forward and reverse primers (pE 5´-AAA CTC AAA GGA ATT GAC GG-3`; pF 5´-ACG AGC TGA CGA CAG CCA TG-3`; Kanto Öqvist *et al*. 2008, J Ind Microbiol Biotechnol 25: 1165–1173). Samples were run in triplicates in 20 µl reactions containing 10 µl 2x PowerUp SYBR Green Master Mix (Thermo scientific, MA, USA), 0.2 µl 20 mg/ml BSA, 0.5 µl of each primer (10 µM), and the sample template. A standard curve was included in every run to allow quantitation of the number of bacterial 16S copies present in the original sample. The q-PCR was run using initial denaturation at 95 °C for 2 min, 40 cycles of denaturation at 95 °C for 10 s, annealing at 53 °C for 20 s, and extension at 72 °C for 30 s. Melting curve analysis on the amplicon was as follows: 95 °C for 10 s, 65 °C for 60 s, 97 °C for 1 s, 37 °C for 30 s with continuous measurement of the fluorescence signal. DNA of *Cupriavidus necator* JMP134 (DSM 4058) was used as the standard and as a a positive control while sterile water was used as a negative control.

## Results

Sand and gravel from aggregate producer had clearly lower abundance of bacterial 16S rDNA copies compared to organic soils and natural forest soils (Table S1). On the contrary, freeze-dried extract contained almost as high amounts of bacterial 16S copies as organic and natural forest soils.

**Supplementary Table 1.** Quantitative PCR results showing the number of bacterial 16S rDNA copies in different kind of soil materials.

| **Material** | **Mean** | **Min** | **Max** | **N** |
| --- | --- | --- | --- | --- |
| Sand/gravel from aggregate producer | 180 000 | 22 000 | 700 000 | 5 |
| Freeze dried extract | 3 500 000 000 | 2 300 000 000 | 4 300 000 000 | 6 |
| Organic soil | 4 200 000 000 | 1 200 000 000 | 12 000 000 000 | 14 |
| Natural forest soil | 4 600 000 000 | 4 300 000 000 | 4 900 000 000 | 2 |

## Supplementary Table 2.

| **Supplementary Table 2. KEGG score** |  |  |  |  |  |  |  |  |
| --- | --- | --- | --- | --- | --- | --- | --- | --- |
| **KEGG functions** | **Before sand + biodiversity blend (mean)** | **SE** | **Before sand**  **(mean)** | **SE** | **After**  **sand + biodiversity blend**  **(mean)** | **SE** | **After sand**  **(mean)** | **SE** |
| Bacterial chemotaxis | 42722.3 | 7454.4 | 28812.8 | 4636.1 | 405362.3 | 22366.1 | 142507.6 | 39544.4 |
| Bacterial motility proteins | 117111.1 | 20584.9 | 79951.8 | 13010.6 | 1059540.3 | 66929.8 | 384844.0 | 107102.6 |
| Cell cycle - Caulobacter | 35203.3 | 6716.3 | 27103.1 | 4776.4 | 356684.5 | 25414.9 | 128730.7 | 39410.6 |
| Cytoskeleton proteins | 18207.7 | 3472.0 | 13728.7 | 2460.4 | 179411.1 | 12475.4 | 67010.1 | 20312.5 |
| Flagellar assembly | 45111.3 | 8102.8 | 30978.9 | 4872.8 | 422501.8 | 25458.8 | 147230.4 | 41359.7 |
| Lysosome | 2584.8 | 580.8 | 1834.9 | 379.4 | 57312.9 | 5723.7 | 14932.2 | 4729.4 |
| Meiosis - yeast | 3549.4 | 633.0 | 2588.3 | 484.3 | 31987.7 | 2753.8 | 15199.4 | 4964.2 |
| Peroxisome | 24225.9 | 5027.6 | 17796.3 | 3390.1 | 236194.8 | 16049.5 | 108874.2 | 34775.6 |
| ABC transporters | 289562.6 | 57315.3 | 215795.3 | 37998.0 | 3400431.6 | 237018.6 | 1317824.4 | 424208.5 |
| Bacterial secretion system | 60519.0 | 11097.4 | 46276.1 | 8136.5 | 492213.2 | 35555.7 | 195871.6 | 56140.8 |
| Bacterial toxins | 6978.2 | 1480.0 | 5200.1 | 991.1 | 69447.6 | 5439.8 | 26983.6 | 8388.6 |
| Cellular antigens | 5738.8 | 1253.1 | 4201.0 | 774.8 | 57724.1 | 4764.3 | 22808.7 | 7003.9 |
| Ion channels | 1880.8 | 403.0 | 1461.8 | 280.4 | 15950.4 | 1119.3 | 8624.5 | 2988.6 |
| MAPK signaling pathway - yeast | 2384.4 | 388.0 | 1792.0 | 295.0 | 21167.6 | 1227.5 | 7187.3 | 1843.4 |
| Phosphatidylinositol signaling system | 8238.8 | 1634.2 | 6383.4 | 1167.3 | 85911.9 | 6583.7 | 30031.0 | 9554.1 |
| Phosphotransferase system (PTS) | 14980.0 | 2722.5 | 13202.1 | 2555.6 | 109907.4 | 5684.1 | 39635.3 | 12969.0 |
| Secretion system | 143107.1 | 25423.3 | 106682.7 | 18075.2 | 1145633.8 | 79485.2 | 447050.3 | 125783.5 |
| Transporters | 479716.0 | 93794.2 | 359284.6 | 63428.7 | 5857608.0 | 410124.4 | 2193838.6 | 716913.9 |
| Two-component system | 166185.6 | 30217.9 | 120056.3 | 20246.8 | 1502125.3 | 92272.3 | 617203.8 | 181470.5 |
| Aminoacyl-tRNA biosynthesis | 78134.5 | 15662.9 | 60219.6 | 11235.2 | 795817.8 | 55294.1 | 296947.6 | 93535.7 |
| Base excision repair | 37448.3 | 8624.9 | 28743.8 | 5688.9 | 395397.3 | 28710.3 | 180174.5 | 59949.9 |
| Chaperones and folding catalysts | 73155.1 | 13708.0 | 54926.8 | 9895.8 | 678344.6 | 50002.3 | 250873.8 | 73306.7 |
| Chromosome | 106695.9 | 19966.6 | 80896.0 | 14198.2 | 915178.8 | 58627.7 | 343372.0 | 101281.2 |
| DNA repair and recombination proteins | 196625.9 | 40191.6 | 150941.8 | 28161.5 | 1930566.9 | 134899.3 | 776807.9 | 246487.8 |
| DNA replication proteins | 71393.4 | 13909.3 | 55143.3 | 10004.4 | 646006.8 | 42059.0 | 246029.6 | 75441.8 |
| DNA replication | 41265.8 | 8312.1 | 32331.2 | 6022.9 | 395739.3 | 26609.1 | 154435.8 | 48470.4 |
| Homologous recombination | 55830.6 | 11222.3 | 43632.5 | 8172.2 | 560011.5 | 40029.9 | 210415.3 | 65863.4 |
| Mismatch repair | 49543.5 | 9978.4 | 38474.3 | 7076.5 | 500255.8 | 35145.2 | 187993.1 | 59136.4 |
| Non-homologous end-joining | 4824.8 | 1208.3 | 3509.3 | 677.4 | 53530.5 | 4183.7 | 32160.8 | 11037.6 |
| Nucleotide excision repair | 26459.3 | 5885.5 | 20227.0 | 3937.0 | 275925.3 | 19644.8 | 121241.8 | 40040.9 |
| Proteasome | 2743.3 | 860.7 | 2090.3 | 542.7 | 31416.5 | 2765.9 | 18649.3 | 6333.9 |
| Protein export | 42475.0 | 8856.3 | 33400.5 | 6309.9 | 434296.8 | 29717.3 | 169282.8 | 54408.0 |
| Protein processing in endoplasmic reticulum | 5622.6 | 1070.3 | 3726.9 | 652.6 | 61477.9 | 5308.1 | 22370.3 | 7092.7 |
| Ribosome biogenesis in eukaryotes | 4441.6 | 997.7 | 3365.8 | 689.4 | 38869.3 | 3058.3 | 18314.2 | 5826.7 |
| Ribosome Biogenesis | 88133.1 | 17539.4 | 68386.3 | 12637.2 | 828041.3 | 55112.1 | 305917.8 | 92175.0 |
| Ribosome | 142846.8 | 28979.8 | 111752.8 | 21041.7 | 1399972.3 | 97087.6 | 526302.3 | 165463.6 |
| RNA degradation | 34197.0 | 6748.7 | 25826.6 | 4714.9 | 322796.5 | 22455.1 | 125319.2 | 38512.9 |
| RNA polymerase | 10993.4 | 2204.2 | 8656.7 | 1619.1 | 97834.2 | 6184.6 | 39286.9 | 12277.7 |
| RNA transport | 6667.8 | 1091.4 | 4984.0 | 802.5 | 72148.8 | 4562.1 | 15090.8 | 3976.9 |
| Sulfur relay system | 28385.8 | 5736.3 | 21363.9 | 3866.8 | 296779.3 | 21344.1 | 115676.5 | 37055.5 |
| Transcription factors | 120271.3 | 22494.7 | 90997.5 | 15872.9 | 1225809.8 | 75004.1 | 473054.9 | 148612.7 |
| Transcription machinery | 57740.8 | 11572.4 | 43818.9 | 8115.5 | 697134.3 | 47278.5 | 272441.3 | 86432.6 |
| Translation factors | 32050.7 | 6445.0 | 24723.3 | 4622.2 | 326995.0 | 24005.3 | 118590.7 | 37092.3 |
| Ubiquitin system | 2530.0 | 685.5 | 2021.1 | 442.6 | 18777.0 | 1382.2 | 11958.5 | 4120.5 |
| African trypanosomiasis | 1004.0 | 321.0 | 860.7 | 211.9 | 18760.3 | 1702.0 | 8744.2 | 3246.4 |
| Alzheimer's disease | 8043.8 | 1381.2 | 6434.1 | 1144.2 | 58239.6 | 4317.9 | 24109.9 | 6512.2 |
| Amoebiasis | 1057.8 | 162.6 | 761.7 | 134.5 | 20507.1 | 1384.7 | 5813.7 | 1785.3 |
| Amyotrophic lateral sclerosis (ALS) | 4822.6 | 914.2 | 3609.3 | 651.8 | 34444.3 | 1884.1 | 12086.3 | 3171.9 |
| Bladder cancer | 1129.0 | 248.2 | 822.6 | 179.3 | 8937.2 | 905.2 | 5547.6 | 1737.2 |
| Chagas disease (American trypanosomiasis) | 924.4 | 311.0 | 785.8 | 198.5 | 16004.5 | 1568.9 | 5710.5 | 2009.1 |
| Epithelial cell signaling in Helicobacter pylori infection | 3581.1 | 613.9 | 2815.5 | 468.2 | 23091.6 | 1237.0 | 7361.6 | 1754.5 |
| Huntington's disease | 8139.8 | 1382.1 | 6206.0 | 1078.6 | 57733.4 | 4589.0 | 21902.9 | 5570.8 |
| Parkinson's disease | 4223.1 | 671.3 | 3283.5 | 581.0 | 24008.1 | 2509.0 | 10912.7 | 2559.1 |
| Pathways in cancer | 4360.4 | 800.1 | 3450.6 | 617.5 | 44308.0 | 3471.1 | 16148.4 | 4674.4 |
| Pertussis | 2234.3 | 370.2 | 1618.0 | 277.3 | 15542.9 | 1522.6 | 5497.1 | 1248.2 |
| Primary immunodeficiency | 3700.2 | 959.5 | 2731.2 | 577.4 | 37393.9 | 2779.3 | 20116.9 | 6767.1 |
| Prostate cancer | 1478.3 | 266.4 | 1070.8 | 187.6 | 15060.2 | 1308.1 | 4687.0 | 1377.0 |
| Renal cell carcinoma | 2141.8 | 457.3 | 1739.2 | 336.5 | 22786.8 | 1631.8 | 8844.8 | 2729.2 |
| Staphylococcus aureus infection | 3645.2 | 599.1 | 3179.8 | 600.3 | 9435.4 | 1778.3 | 2313.3 | 645.8 |
| Tuberculosis | 11920.1 | 2362.0 | 8947.0 | 1640.1 | 120835.5 | 9133.8 | 45514.9 | 14036.9 |
| Type I diabetes mellitus | 3881.1 | 838.3 | 2957.1 | 602.3 | 45017.3 | 3480.3 | 19047.2 | 6205.1 |
| Type II diabetes mellitus | 2979.8 | 591.1 | 2201.0 | 401.0 | 30662.7 | 2327.4 | 10216.7 | 3142.4 |
| Vibrio cholerae pathogenic cycle | 5668.6 | 971.9 | 4129.0 | 702.1 | 47302.0 | 2873.0 | 21102.5 | 6178.1 |
| Alanine, aspartate and glutamate metabolism | 71712.5 | 14859.5 | 54074.3 | 10066.9 | 738399.4 | 53736.4 | 319319.7 | 104259.3 |
| alpha-Linolenic acid metabolism | 2963.0 | 638.9 | 2114.3 | 400.4 | 40184.3 | 2829.4 | 20106.7 | 6824.8 |
| Amino acid related enzymes | 98765.4 | 19765.1 | 75254.3 | 13982.2 | 1014358.4 | 71487.5 | 382704.8 | 119927.4 |
| Amino sugar and nucleotide sugar metabolism | 75621.8 | 16056.3 | 57539.2 | 10748.0 | 909931.5 | 67513.7 | 343869.6 | 112682.8 |
| Aminobenzoate degradation | 43706.3 | 8181.4 | 32013.1 | 5736.8 | 376998.2 | 28019.4 | 210442.2 | 66874.5 |
| Arachidonic acid metabolism | 6496.3 | 1248.6 | 4662.3 | 765.6 | 54572.6 | 4243.8 | 23805.1 | 7146.9 |
| Arginine and proline metabolism | 93712.3 | 18086.6 | 69580.3 | 12389.3 | 1058059.0 | 76755.1 | 436194.3 | 140261.6 |
| Ascorbate and aldarate metabolism | 16905.5 | 2991.8 | 12318.5 | 2106.0 | 135864.5 | 8860.8 | 65237.4 | 20494.1 |
| Atrazine degradation | 6287.3 | 1254.2 | 4595.8 | 883.0 | 51741.5 | 4466.7 | 25349.7 | 7988.7 |
| Benzoate degradation | 60331.2 | 11162.3 | 43453.3 | 7734.6 | 497646.5 | 34401.2 | 286157.9 | 93015.1 |
| beta-Alanine metabolism | 43492.8 | 8843.1 | 31686.6 | 5791.4 | 391731.7 | 29092.3 | 223746.6 | 71088.2 |
| beta-Lactam resistance | 3366.4 | 581.9 | 2439.9 | 397.8 | 24278.3 | 1949.6 | 9732.8 | 2815.7 |
| Biosynthesis of ansamycins | 4811.9 | 904.8 | 3531.3 | 629.0 | 50708.9 | 3860.0 | 17477.0 | 5613.3 |
| Biosynthesis of siderophore group nonribosomal peptides | 3862.0 | 839.7 | 3234.8 | 608.0 | 43886.8 | 2908.2 | 20339.0 | 6699.0 |
| Biosynthesis of unsaturated fatty acids | 21712.7 | 4164.9 | 15646.0 | 2720.7 | 220589.9 | 16573.5 | 97171.7 | 29836.7 |
| Biosynthesis of vancomycin group antibiotics | 4388.8 | 1010.7 | 2942.7 | 587.6 | 44361.0 | 3738.0 | 18247.6 | 5756.9 |
| Biotin metabolism | 11307.2 | 2192.1 | 8668.8 | 1625.0 | 87962.1 | 6180.1 | 39340.0 | 11401.2 |
| Bisphenol degradation | 9099.1 | 1649.8 | 7238.5 | 1329.2 | 93302.3 | 6900.3 | 52583.8 | 16653.3 |
| Butanoate metabolism | 92524.6 | 17977.2 | 67772.8 | 12141.1 | 904437.1 | 66089.3 | 461200.1 | 149217.6 |
| Butirosin and neomycin biosynthesis | 3228.8 | 797.5 | 2447.6 | 485.8 | 55522.7 | 4906.6 | 21055.7 | 7217.9 |
| C5-Branched dibasic acid metabolism | 26236.9 | 5129.5 | 19665.5 | 3498.8 | 279076.8 | 21838.6 | 104845.8 | 33668.2 |
| Caprolactam degradation | 22445.6 | 4572.1 | 16433.8 | 3086.0 | 208417.6 | 17264.1 | 134808.4 | 43797.5 |
| Carbon fixation in photosynthetic organisms | 37245.1 | 7261.7 | 27875.8 | 4982.5 | 401038.8 | 28870.6 | 142418.3 | 44636.5 |
| Carbon fixation pathways in prokaryotes | 87400.0 | 17231.9 | 64868.2 | 11540.1 | 898561.8 | 64605.6 | 369178.8 | 116614.4 |
| Carotenoid biosynthesis | 4690.3 | 762.5 | 3662.4 | 571.5 | 47083.6 | 3965.4 | 11223.5 | 3084.8 |
| Chloroalkane and chloroalkene degradation | 26226.6 | 4553.5 | 19232.6 | 3221.7 | 227215.0 | 14456.0 | 105186.3 | 32301.8 |
| Chlorocyclohexane and chlorobenzene degradation | 13437.7 | 2242.8 | 9325.8 | 1544.4 | 82497.3 | 6777.2 | 44677.0 | 13806.8 |
| Citrate cycle (TCA cycle | 69132.1 | 13830.9 | 51521.9 | 9399.7 | 729437.0 | 55372.6 | 316273.8 | 102739.0 |
| Cyanoamino acid metabolism | 17559.0 | 3319.2 | 12938.8 | 2266.9 | 195071.2 | 15175.6 | 85137.2 | 28143.4 |
| Cysteine and methionine metabolism | 64743.0 | 12936.8 | 49336.1 | 8988.6 | 718539.1 | 49142.6 | 266520.1 | 85188.5 |
| D-Alanine metabolism | 8093.8 | 1483.7 | 6417.7 | 1094.3 | 73941.7 | 4486.1 | 30757.2 | 9710.7 |
| D-Glutamine and D-glutamate metabolism | 9801.5 | 1982.4 | 7609.4 | 1423.3 | 93734.5 | 6315.9 | 36744.8 | 11594.5 |
| Dioxin degradation | 5257.8 | 833.9 | 4198.4 | 698.9 | 44389.9 | 3400.9 | 25522.0 | 8107.8 |
| Drug metabolism - cytochrome P450 | 19174.2 | 3162.9 | 13976.0 | 2246.7 | 129124.7 | 9816.4 | 57323.1 | 15669.5 |
| Drug metabolism - other enzymes | 18538.8 | 4202.1 | 14112.3 | 2774.7 | 220196.6 | 16206.3 | 89544.1 | 30196.0 |
| Ether lipid metabolism | 2605.8 | 527.4 | 1638.1 | 323.4 | 11781.1 | 1205.9 | 7960.7 | 2450.4 |
| Ethylbenzene degradation | 8673.6 | 1660.9 | 6519.0 | 1141.2 | 74019.3 | 4422.0 | 36213.1 | 11451.6 |
| Fatty acid biosynthesis | 43760.5 | 7893.1 | 32508.9 | 5419.5 | 461665.3 | 30911.2 | 166703.1 | 50409.0 |
| Fatty acid metabolism | 68786.3 | 13884.0 | 50497.5 | 9245.1 | 657568.7 | 47588.0 | 358708.4 | 114752.1 |
| Flavonoid biosynthesis | 1286.8 | 283.9 | 852.3 | 182.3 | 24791.2 | 1796.0 | 10011.7 | 3330.9 |
| Fluorobenzoate degradation | 7543.0 | 1329.1 | 5058.3 | 859.1 | 40899.7 | 3507.5 | 28257.6 | 8908.8 |
| Folate biosynthesis | 37671.3 | 7573.8 | 28756.5 | 5245.3 | 359546.0 | 24884.7 | 140614.7 | 43694.4 |
| Fructose and mannose metabolism | 40342.5 | 8486.5 | 30739.4 | 5748.4 | 505270.3 | 37399.0 | 202013.2 | 67848.4 |
| Galactose metabolism | 28529.0 | 6345.4 | 22763.2 | 4276.3 | 414687.8 | 33366.3 | 145570.8 | 48701.6 |
| Geraniol degradation | 33108.7 | 6815.9 | 24210.4 | 4550.1 | 309744.6 | 22784.8 | 188929.0 | 60433.5 |
| Glutathione metabolism | 38021.5 | 7078.9 | 27875.1 | 4808.4 | 278790.5 | 21739.4 | 121940.0 | 35022.9 |
| Glycerolipid metabolism | 29000.1 | 5719.8 | 21903.2 | 3843.8 | 360679.0 | 27188.7 | 128517.7 | 42355.5 |
| Glycerophospholipid metabolism | 42745.8 | 8358.7 | 31635.8 | 5648.4 | 405939.3 | 26896.1 | 149543.8 | 46186.8 |
| Glycine, serine and threonine metabolism | 75911.8 | 15180.9 | 57528.7 | 10336.2 | 775426.2 | 55684.8 | 324295.8 | 102638.2 |
| Glycolysis / Gluconeogenesis | 89350.1 | 17904.5 | 68317.4 | 12284.2 | 987144.8 | 71450.0 | 403897.3 | 131494.0 |
| Glycosaminoglycan degradation | 1135.7 | 327.9 | 828.9 | 182.0 | 27241.8 | 2996.1 | 6708.8 | 1973.1 |
| Glycosphingolipid biosynthesis - ganglio series | 663.5 | 188.5 | 513.1 | 120.7 | 14233.3 | 1580.5 | 2849.3 | 763.9 |
| Glycosphingolipid biosynthesis - globo series | 1671.4 | 474.0 | 1278.2 | 270.5 | 63953.2 | 6410.6 | 13183.8 | 4517.8 |
| Glycosyltransferases | 27276.6 | 5274.8 | 20897.5 | 3845.1 | 268144.9 | 21791.5 | 103263.9 | 31541.4 |
| Glyoxylate and dicarboxylate metabolism | 68192.0 | 12738.6 | 49248.2 | 8455.6 | 615991.3 | 42701.8 | 278240.4 | 86625.1 |
| Histidine metabolism | 46929.0 | 9430.3 | 35272.4 | 6548.3 | 516231.3 | 34580.2 | 201076.4 | 64553.0 |
| Inositol phosphate metabolism | 17864.0 | 3883.3 | 12647.9 | 2375.3 | 205588.2 | 14298.7 | 80010.9 | 26318.0 |
| Isoquinoline alkaloid biosynthesis | 4144.0 | 722.7 | 2964.5 | 517.5 | 60811.9 | 4620.7 | 22735.2 | 7749.7 |
| Limonene and pinene degradation | 36147.5 | 7020.2 | 27075.2 | 4859.0 | 313067.5 | 22618.8 | 187167.8 | 59079.3 |
| Linoleic acid metabolism | 4313.9 | 885.5 | 3266.7 | 615.8 | 45918.3 | 3338.9 | 24810.4 | 8160.6 |
| Lipid biosynthesis proteins | 63928.7 | 12360.4 | 46890.5 | 8422.1 | 698142.6 | 49165.7 | 276648.9 | 86194.5 |
| Lipoic acid metabolism | 6701.9 | 1309.9 | 5113.3 | 929.3 | 59787.5 | 4047.3 | 24252.9 | 7751.6 |
| Lipopolysaccharide biosynthesis proteins | 33139.4 | 6112.7 | 23221.0 | 3961.0 | 238165.4 | 20376.5 | 85199.3 | 23009.8 |
| Lipopolysaccharide biosynthesis | 24511.7 | 4612.0 | 16941.7 | 2904.6 | 172873.4 | 15228.5 | 65299.1 | 18260.5 |
| Lysine biosynthesis | 46621.1 | 9339.3 | 35369.3 | 6563.9 | 504759.4 | 36624.8 | 185855.8 | 58377.8 |
| Lysine degradation | 46037.3 | 9337.2 | 34130.8 | 6242.0 | 425171.6 | 30204.2 | 233403.8 | 74371.0 |
| Metabolism of xenobiotics by cytochrome P450 | 18641.2 | 3086.2 | 13540.8 | 2173.5 | 118090.3 | 9096.8 | 51946.8 | 13716.3 |
| Methane metabolism | 80723.0 | 16282.3 | 59393.2 | 10872.7 | 951168.6 | 70836.6 | 370100.5 | 121712.5 |
| Naphthalene degradation | 27452.1 | 5032.5 | 20876.3 | 3626.9 | 211623.3 | 13105.8 | 105158.1 | 32153.2 |
| N-Glycan biosynthesis | 2268.6 | 605.3 | 1605.3 | 364.9 | 55226.3 | 5778.6 | 16067.0 | 5729.5 |
| Nicotinate and nicotinamide metabolism | 32874.2 | 6542.2 | 24524.5 | 4538.3 | 328131.0 | 23772.5 | 137541.2 | 43957.3 |
| Nitrogen metabolism | 64845.9 | 12742.2 | 48336.3 | 8645.3 | 564968.3 | 40174.8 | 251352.3 | 77736.7 |
| Nitrotoluene degradation | 6199.3 | 1338.1 | 4106.2 | 816.1 | 94645.7 | 9874.7 | 48352.4 | 17127.1 |
| Novobiocin biosynthesis | 10243.0 | 2043.8 | 7713.9 | 1434.9 | 116041.6 | 8426.7 | 43299.2 | 14021.0 |
| One carbon pool by folate | 37078.0 | 7562.5 | 28715.6 | 5365.9 | 382167.9 | 26659.2 | 146032.2 | 45947.3 |
| Other glycan degradation | 4027.9 | 971.0 | 3052.4 | 636.4 | 92105.2 | 8264.3 | 23353.8 | 7863.9 |
| Oxidative phosphorylation | 111026.6 | 22182.8 | 81638.2 | 14933.8 | 1106249.4 | 86952.3 | 414433.8 | 126131.6 |
| Pantothenate and CoA biosynthesis | 47441.8 | 9201.4 | 35749.3 | 6357.8 | 456414.9 | 32683.4 | 181459.6 | 57391.5 |
| Penicillin and cephalosporin biosynthesis | 4694.3 | 948.4 | 3303.3 | 580.2 | 66664.4 | 5652.2 | 21835.4 | 7211.8 |
| Pentose and glucuronate interconversions | 31229.1 | 5991.6 | 23391.3 | 3984.9 | 435375.9 | 34348.8 | 164667.6 | 55276.8 |
| Pentose phosphate pathway | 55940.8 | 11251.4 | 42069.5 | 7560.4 | 632186.7 | 46369.1 | 241226.2 | 77814.9 |
| Peptidases | 114553.8 | 23672.4 | 85551.4 | 15791.5 | 1266213.8 | 89610.1 | 474019.3 | 150635.3 |
| Peptidoglycan biosynthesis | 52908.9 | 10607.6 | 40914.0 | 7426.9 | 488511.6 | 32281.2 | 193463.6 | 59329.5 |
| Phenylalanine metabolism | 33086.1 | 6221.7 | 23506.2 | 4162.9 | 336227.8 | 26842.5 | 169179.9 | 56484.0 |
| Phenylalanine, tyrosine and tryptophan biosynthesis | 56839.7 | 11337.6 | 43162.6 | 7927.9 | 586026.5 | 41530.7 | 217139.8 | 68337.9 |
| Phenylpropanoid biosynthesis | 5948.3 | 1264.2 | 4271.0 | 746.1 | 105625.2 | 8395.7 | 44970.0 | 15928.7 |
| Phosphonate and phosphinate metabolism | 5174.3 | 875.5 | 3791.8 | 630.1 | 42508.6 | 2545.2 | 11719.2 | 2959.9 |
| Photosynthesis proteins | 25542.7 | 5117.1 | 19393.4 | 3542.4 | 237233.3 | 16123.9 | 87702.5 | 27015.5 |
| Photosynthesis | 23270.8 | 4714.1 | 17787.0 | 3311.7 | 222825.5 | 15565.3 | 81629.9 | 25315.6 |
| Polycyclic aromatic hydrocarbon degradation | 13815.8 | 2454.1 | 10918.6 | 2002.3 | 137282.7 | 9558.9 | 73710.2 | 24581.1 |
| Polyketide sugar unit biosynthesis | 12251.5 | 2741.1 | 8449.3 | 1651.6 | 117347.7 | 9717.8 | 43953.7 | 13166.2 |
| Porphyrin and chlorophyll metabolism | 68954.3 | 14602.8 | 52104.5 | 9953.5 | 733319.4 | 49811.8 | 280662.2 | 87245.3 |
| Prenyltransferases | 25812.4 | 5419.8 | 19992.1 | 3808.5 | 279147.1 | 19788.5 | 105152.9 | 33995.2 |
| Primary bile acid biosynthesis | 1636.8 | 379.3 | 1293.9 | 225.8 | 16245.7 | 1591.3 | 9434.4 | 2885.9 |
| Propanoate metabolism | 85460.2 | 16831.5 | 62942.0 | 11125.8 | 821384.9 | 57673.4 | 412853.6 | 131019.0 |
| Protein kinases | 33089.3 | 6256.4 | 24466.2 | 4269.6 | 323103.3 | 19327.5 | 136740.2 | 41903.5 |
| Purine metabolism | 163126.6 | 33053.0 | 124949.8 | 23320.7 | 1539370.5 | 108598.6 | 619339.5 | 194320.6 |
| Pyrimidine metabolism | 108039.3 | 21952.6 | 83751.6 | 15647.7 | 1081781.3 | 73442.5 | 422491.9 | 133830.8 |
| Pyruvate metabolism | 100299.4 | 18594.6 | 75600.2 | 12953.9 | 969606.8 | 67843.3 | 405693.6 | 127733.0 |
| Retinol metabolism | 9266.5 | 1648.3 | 6908.2 | 1174.5 | 77593.1 | 5447.0 | 32828.7 | 9867.6 |
| Riboflavin metabolism | 20199.3 | 4054.6 | 15491.8 | 2820.9 | 186934.4 | 12195.7 | 89933.7 | 28479.3 |
| Selenocompound metabolism | 29181.0 | 5919.3 | 22241.1 | 4048.6 | 313848.5 | 21722.0 | 113899.0 | 35354.5 |
| Sphingolipid metabolism | 4869.3 | 1102.8 | 3430.2 | 682.7 | 105785.7 | 10187.6 | 24217.9 | 8081.6 |
| Starch and sucrose metabolism | 43407.4 | 9321.9 | 32987.8 | 6355.2 | 562605.4 | 42642.4 | 214533.8 | 72429.9 |
| Steroid biosynthesis | 1646.8 | 338.6 | 1075.3 | 210.8 | 20961.3 | 1388.8 | 7198.9 | 2230.6 |
| Steroid hormone biosynthesis | 1867.0 | 383.5 | 1488.2 | 296.1 | 26190.1 | 2334.2 | 12087.8 | 4258.1 |
| Stilbenoid, diarylheptanoid and gingerol biosynthesis | 1693.3 | 324.3 | 1569.1 | 402.2 | 34011.9 | 3249.8 | 21964.0 | 7271.8 |
| Streptomycin biosynthesis | 22089.4 | 5177.6 | 15814.8 | 3152.3 | 264449.9 | 22076.6 | 100028.8 | 32033.1 |
| Styrene degradation | 9272.7 | 1626.9 | 6530.7 | 1155.7 | 88678.9 | 7793.5 | 49133.8 | 16389.1 |
| Sulfur metabolism | 28347.4 | 5422.5 | 21501.2 | 3782.0 | 245264.5 | 17421.0 | 98811.5 | 29693.6 |
| Synthesis and degradation of ketone bodies | 16363.4 | 3264.2 | 11998.7 | 2133.5 | 153341.1 | 9643.8 | 76309.7 | 23917.7 |
| Taurine and hypotaurine metabolism | 10044.1 | 2047.2 | 7620.0 | 1338.3 | 94799.9 | 7194.6 | 43326.0 | 13849.8 |
| Terpenoid backbone biosynthesis | 42664.6 | 8835.3 | 32818.3 | 6122.4 | 424622.9 | 29078.9 | 180641.4 | 58040.9 |
| Tetracycline biosynthesis | 12400.5 | 2029.1 | 9459.4 | 1476.2 | 101207.9 | 6089.3 | 30558.5 | 8059.3 |
| Thiamine metabolism | 29652.8 | 5629.4 | 23194.3 | 4040.2 | 294815.8 | 19400.0 | 112799.6 | 35452.4 |
| Toluene degradation | 20634.2 | 3827.5 | 14841.3 | 2658.1 | 157653.4 | 12834.9 | 80191.0 | 26090.8 |
| Tropane, piperidine and pyridine alkaloid biosynthesis | 9681.9 | 1963.0 | 7268.3 | 1373.9 | 117224.3 | 8415.2 | 47317.3 | 15383.8 |
| Tryptophan metabolism | 57391.3 | 11237.7 | 42030.8 | 7572.1 | 556494.9 | 40080.0 | 296353.5 | 96288.1 |
| Tyrosine metabolism | 43018.0 | 8161.1 | 31754.8 | 5721.5 | 398471.8 | 27058.7 | 178826.3 | 58712.6 |
| Ubiquinone and other terpenoid-quinone biosynthesis | 28895.7 | 5703.2 | 21594.3 | 3981.2 | 294854.0 | 21712.4 | 111264.5 | 35199.2 |
| Valine, leucine and isoleucine biosynthesis | 61743.7 | 12369.4 | 46696.7 | 8469.4 | 653196.3 | 50551.1 | 260031.9 | 83230.3 |
| Valine, leucine and isoleucine degradation | 79503.7 | 16007.5 | 58838.1 | 10597.3 | 797253.6 | 55888.6 | 424858.9 | 135022.3 |
| Vitamin B6 metabolism | 13617.9 | 2844.8 | 10320.8 | 1926.3 | 151977.5 | 11403.3 | 60013.9 | 19480.9 |
| Xylene degradation | 4414.8 | 773.8 | 3525.2 | 648.7 | 52475.3 | 5031.1 | 31539.8 | 11130.9 |
| Zeatin biosynthesis | 2649.8 | 541.9 | 2072.6 | 393.6 | 26513.6 | 1877.5 | 9893.4 | 3119.9 |
| Adipocytokine signaling pathway | 7838.3 | 1720.5 | 5723.8 | 1190.1 | 93557.2 | 6947.4 | 47604.1 | 16308.2 |
| Antigen processing and presentation | 1478.3 | 266.4 | 1070.8 | 187.6 | 15060.2 | 1308.1 | 4687.0 | 1377.0 |
| Cardiac muscle contraction | 3484.9 | 558.5 | 2643.8 | 471.5 | 17967.7 | 1903.9 | 8347.5 | 1973.9 |
| Circadian rhythm - plant | 668.4 | 193.4 | 413.0 | 111.6 | 10525.2 | 634.0 | 5687.6 | 1980.6 |
| Glutamatergic synapse | 5900.7 | 1420.5 | 4548.3 | 935.7 | 71660.4 | 5360.1 | 34897.5 | 11480.0 |
| Insulin signaling pathway | 4731.5 | 1003.1 | 3375.2 | 677.4 | 53621.9 | 4083.6 | 22592.3 | 7714.9 |
| Mineral absorption | 1666.8 | 258.5 | 1339.5 | 215.0 | 11774.3 | 609.7 | 5069.1 | 1393.5 |
| NOD-like receptor signaling pathway | 1499.7 | 270.5 | 1103.9 | 197.5 | 15092.8 | 1308.4 | 4698.8 | 1375.8 |
| Plant-pathogen interaction | 10558.8 | 1948.7 | 7798.8 | 1338.9 | 101828.5 | 7429.8 | 35194.8 | 10455.9 |
| PPAR signaling pathway | 18146.9 | 4093.6 | 13270.6 | 2653.9 | 189395.2 | 14810.0 | 101579.6 | 33049.0 |
| Progesterone-mediated oocyte maturation | 1478.3 | 266.4 | 1070.8 | 187.6 | 15060.2 | 1308.1 | 4687.0 | 1377.0 |
| Proximal tubule bicarbonate reclamation | 2868.5 | 637.5 | 2140.2 | 459.4 | 37975.3 | 3313.8 | 16192.4 | 5680.4 |
| Amino acid metabolism | 18249.8 | 3321.8 | 13208.5 | 2277.4 | 214474.4 | 12954.0 | 86649.7 | 28743.2 |
| Biosynthesis and biodegradation of secondary metabolites | 6574.3 | 1376.7 | 4919.8 | 951.2 | 54238.3 | 3506.9 | 30673.2 | 10176.5 |
| Carbohydrate metabolism | 8391.2 | 1869.8 | 6143.8 | 1204.3 | 122743.4 | 9039.1 | 45571.3 | 15389.0 |
| Cell division | 5743.4 | 1027.6 | 4287.8 | 756.2 | 48576.0 | 3610.8 | 16646.5 | 4739.2 |
| Cell motility and secretion | 18915.3 | 3396.8 | 13239.2 | 2331.5 | 169568.5 | 13822.9 | 59026.0 | 16741.0 |
| Electron transfer carriers | 1927.8 | 550.2 | 1457.5 | 333.8 | 29381.2 | 1707.3 | 14811.3 | 5448.1 |
| Energy metabolism | 65396.8 | 12515.2 | 47312.1 | 8610.5 | 682189.8 | 49050.8 | 274116.3 | 84507.5 |
| Function unknown | 128562.1 | 24509.0 | 95776.0 | 17152.6 | 1247946.4 | 84142.8 | 460860.4 | 140649.2 |
| General function prediction only | 283707.2 | 54914.6 | 213304.4 | 37598.9 | 2990218.9 | 208693.1 | 1144532.3 | 355161.8 |
| Germination | 494.1 | 106.4 | 448.8 | 98.2 | 27726.4 | 4239.5 | 317.6 | 79.0 |
| Glycan biosynthesis and metabolism | 4876.4 | 840.1 | 3391.9 | 571.1 | 31583.6 | 2760.2 | 9897.9 | 2397.7 |
| Inorganic ion transport and metabolism | 28851.0 | 5460.8 | 21084.4 | 3763.3 | 266511.3 | 20565.4 | 107525.8 | 31653.5 |
| Lipid metabolism | 11176.9 | 1926.0 | 7788.1 | 1332.3 | 78712.2 | 5414.5 | 32665.3 | 8930.1 |
| Membrane and intracellular structural molecules | 50457.3 | 8735.5 | 37182.4 | 6448.7 | 378997.8 | 31215.6 | 140144.7 | 36620.7 |
| Metabolism of cofactors and vitamins | 16215.9 | 3043.9 | 11818.0 | 2069.2 | 149531.7 | 10148.9 | 62023.7 | 19099.7 |
| Nucleotide metabolism | 3412.7 | 776.2 | 2564.5 | 502.3 | 33816.8 | 2908.1 | 11412.1 | 3994.3 |
| Other ion-coupled transporters | 108514.8 | 20418.2 | 83112.8 | 14782.9 | 869741.3 | 47105.7 | 308657.3 | 94042.6 |
| Other transporters | 18242.2 | 3258.4 | 13908.3 | 2405.2 | 161385.9 | 11928.6 | 56840.8 | 16052.3 |
| Others | 83002.5 | 15489.0 | 62086.4 | 10993.8 | 831847.1 | 53757.9 | 344221.3 | 109984.5 |
| Pores ion channels | 35459.7 | 6081.9 | 26333.9 | 4487.0 | 248017.2 | 20658.7 | 95869.6 | 23978.8 |
| Protein folding and associated processing | 59912.1 | 12115.3 | 44995.5 | 8371.5 | 625322.6 | 48151.6 | 226060.8 | 69147.2 |
| Replication, recombination and repair proteins | 66017.4 | 11773.3 | 50931.9 | 8739.7 | 548055.0 | 39427.4 | 227081.3 | 69572.7 |
| Restriction enzyme | 7636.7 | 1414.9 | 5755.2 | 1087.4 | 85525.5 | 6920.6 | 27426.4 | 8548.8 |
| Signal transduction mechanisms | 37970.8 | 6843.1 | 28098.4 | 4773.3 | 337077.3 | 21014.2 | 129243.5 | 37608.2 |
| Sporulation | 4129.8 | 841.7 | 3422.1 | 623.5 | 169033.3 | 14715.5 | 13640.7 | 4331.6 |
| Translation proteins | 58861.1 | 11264.9 | 44727.5 | 8007.9 | 575556.9 | 41289.9 | 190739.9 | 56113.9 |
